# Tackling unbalanced datasets for yellow and brown rust detection in wheat

**DOI:** 10.3389/fpls.2024.1392409

**Published:** 2024-05-14

**Authors:** Carmen Cuenca-Romero, Orly Enrique Apolo-Apolo, Jaime Nolasco Rodríguez Vázquez, Gregorio Egea, Manuel Pérez-Ruiz

**Affiliations:** ^1^Universidad de Sevilla, Área de Ingeniería Agroforestal, Dpto. de Ingeniería Aeroespacial y Mecánica de Fluidos, Seville, Spain; ^2^Department of Earth and Environmental Sciences, KU Leuven, Leuven, Belgium

**Keywords:** wheat, rust, SMOTE, unbalanced datasets, machine learning

## Abstract

This study evaluates the efficacy of hyperspectral data for detecting yellow and brown rust in wheat, employing machine learning models and the SMOTE (Synthetic Minority Oversampling Technique) augmentation technique to tackle unbalanced datasets. Artificial Neural Network (ANN), Support Vector Machine (SVM), Random Forest (RF), and Gaussian Naïve Bayes (GNB) models were assessed. Overall, SVM and RF models showed higher accuracies, particularly when utilizing SMOTE-enhanced datasets. The RF model achieved 70% accuracy in detecting yellow rust without data alteration. Conversely, for brown rust, the SVM model outperformed others, reaching 63% accuracy with SMOTE applied to the training set. This study highlights the potential of spectral data and machine learning (ML) techniques in plant disease detection. It emphasizes the need for further research in data processing methodologies, particularly in exploring the impact of techniques like SMOTE on model performance.

## 1 Introduction

In wheat crop, foliar diseases such as rust are directly related to decreased yield and grain quality (Figueroa et al., 2018). Yield losses caused by diseases depend on the crop cultivar’s resistance or susceptibility and the specific type of rust affecting the crop. Potential losses may reach up to 5% in resistant varieties, but in highly susceptible cultivars, this value can be 80% under favorable conditions for the disease (Beard et al., 2005). To reduce the effects of the disease on yield, farmers often make preventive applications when the first pustules are seen. However, treatments are usually not effective since the damage caused by the rust has already occurred at the cellular level (Bauriegel and Herppich, 2014). Consequently, early disease detection is essential to optimize their management and maximize crop production (Salvagiotti et al., 2005; Orchi et al., 2021).

As mentioned, crop disease identification primarily relies on human visual inspection (Yadav et al., 2019). However, this method is subjective, time-consuming, and prone to human error (Bock et al., 2010). As an alternative to visual methods, many technologies based on remote sensing have been developed to achieve more accurate, rapid, and cost-effective detection of crop diseases (Zhang et al., 2019). These technologies offer great potential for early and non-destructive detection of plant diseases, enabling timely intervention (Terentev et al., 2022).

From a remote-sensing perspective, disease detection uses various tools (Yang, 2020). However, in the past years, spectral information has gained significance, as highlighted by Wan et al. (2022). The reliance on spectral information is based on the understanding that each disease induces unique spectral reflectivity patterns in crops, resulting from the harm inflicted on plant tissues (Clevers, 1999). The changes can be detected by hyperspectral sensors (spectroradiometers and cameras), which are considered state-of-the-art for disease detection in crops (Khanal et al., 2020). These sensors offer an exceptional level of spectral resolution, capturing data related to biotic and abiotic stresses that might not be easily detected by other sensors with lower spectral resolution (Weiss et al., 2020). Given this scenario, hyperspectral cameras have emerged as a promising alternative to spectroradiometers among the hyperspectral sensors. They provide the unique capability to capture a high-resolution spectrum for each pixel in an image. Despite their advantages, hyperspectral images have several limitations, independent of the equipment’s cost, that should be considered. According to the work conducted by Roberts et al. (2018), the issues are related to the availability of robust commercial instrumentation and the large amount of data generated during the analysis. Due to the large amount of data generated, hyperspectral images require extensive processing work, which involves a significant amount of time and complex algorithms to reduce spectral dimensionality (Paoletti et al., 2019). In this regard, spectroradiometers offer less detailed information because they do not produce an image as an outcome. However, they are a more affordable solution in terms of equipment cost and data processing.

Many approaches have been derived for data processing of spectroradiometers and hyperspectral cameras. One of the most widely used approaches is the application of vegetation spectral indices obtained from the combination of specific spectral bands (Giovos et al., 2021). These indices may detect crop diseases by observing changes in the leaf’s external (i.e., necrosis and chlorosis) and internal architecture (i.e., chloroplast dysfunction), as explained by Lin et al. (2017). Extensive research has been conducted to detect diseases using spectral indices. As an example, Devadas et al. (2009) showed the suitability of specific indices like the Anthocyanin Reflectance Index (ARI) to discriminate between healthy and rust-infected wheat leaves at a medium-late growth stage and the Transformed Chlorophyll Absorption in Reflectance Index (TCARI) to detect wheat leaf rust. Other studies, such as the one conducted by Ashourloo et al. (2014), demonstrated remarkable accuracies exceeding 85% in estimating disease severity using a Leaf Rust Disease Severity Index (LRDSI). While the LRDSI has been successful, it has limitations in the early detection of symptoms due to the spectral similarity between affected and healthy leaf areas. Spectral indices offer valuable insights; however, they may fall short in specific scenarios as they don’t encompass the comprehensive data required for in-depth research analysis.

An alternative to employing spectral indices for disease identification is leveraging the full spectrum of radiation reflected and captured by hyperspectral sensors. However, given the vastness of hyperspectral datasets and their intricate processing requirements, integrating ML models with hyperspectral data for disease identification has garnered increased interest in recent years. In this sense, models such as ANN, SVM, RF, and GNB, among others, have been proposed (Singh et al., 2016; Su, 2020). In light of these facts, hyperspectral information for disease detection has been successfully utilized. However, the research often relies on datasets with limited data volume, particularly concerning the context of ML. A comprehensive and balanced dataset is essential for broad generalization when constructing a resilient ML model. However, field data collection requires considerable effort and resources, which limits data availability for analysis. Because of this, data augmentation techniques are expected to be employed to improve the overall learning procedure and performance of ML models. Data augmentation is primarily performed on imbalanced datasets, which exhibit a significant disparity in the number of data instances in each class (Hadad et al., 2009). This imbalance has consequences for the learning process by resulting in low predictive accuracy for the minority class (Daskalaki et al., 2006), as many performance measures used to guide training penalize minority classes. Rules that predict minority classes are highly specialized and have low coverage, which often causes them to be discarded in favor of more general rules. In addition, the noise treatment may affect the classification of minority classes, as they may be erroneously discarded as noise (Pulgar et al., 2017).

According to the literature review by Kamilaris and Prenafeta-Boldú (2018), 37% of the reviewed articles apply data augmentation and highlight the importance of such techniques in scientific works with small hyperspectral datasets (i.e., images). Limited resources are available concerning the refinement of hyperspectral data from spectroscopy. Chawla et al. (2002) introduced the Synthetic Minority Over-sampling Technique (SMOTE), which interpolates between minority class instances to address data imbalance. This tool augments the minority class by generating new synthetic data based on existing examples. From an agriculture perspective, researchers like Ma et al. (2019) employed SMOTE to balance the imbalanced training dataset, aiming to develop a model that distinguishes between powdery mildew and aphid infestations in winter wheat using bi-temporal Landsat-8 imagery. A recent study by Divakar et al. (2021) utilized SMOTE to classify areas affected by wilt disease in bananas.

Based on the above literature review and our knowledge, this technique has rarely been applied to agricultural tasks, particularly for detecting wheat yellow and brown rust. Hence, this study aims to evaluate the feasibility of differentiating cultivars affected by yellow and brown rust in durum and bread wheat using complete spectral signatures acquired through spectroscopy. Moreover, it will assess the impact of the SMOTE algorithm on the development of ML models for the accurate detection of both types of rust.

## 2 Materials and methods

### 2.1 Field experiment and data acquisition

**Figure 1:**
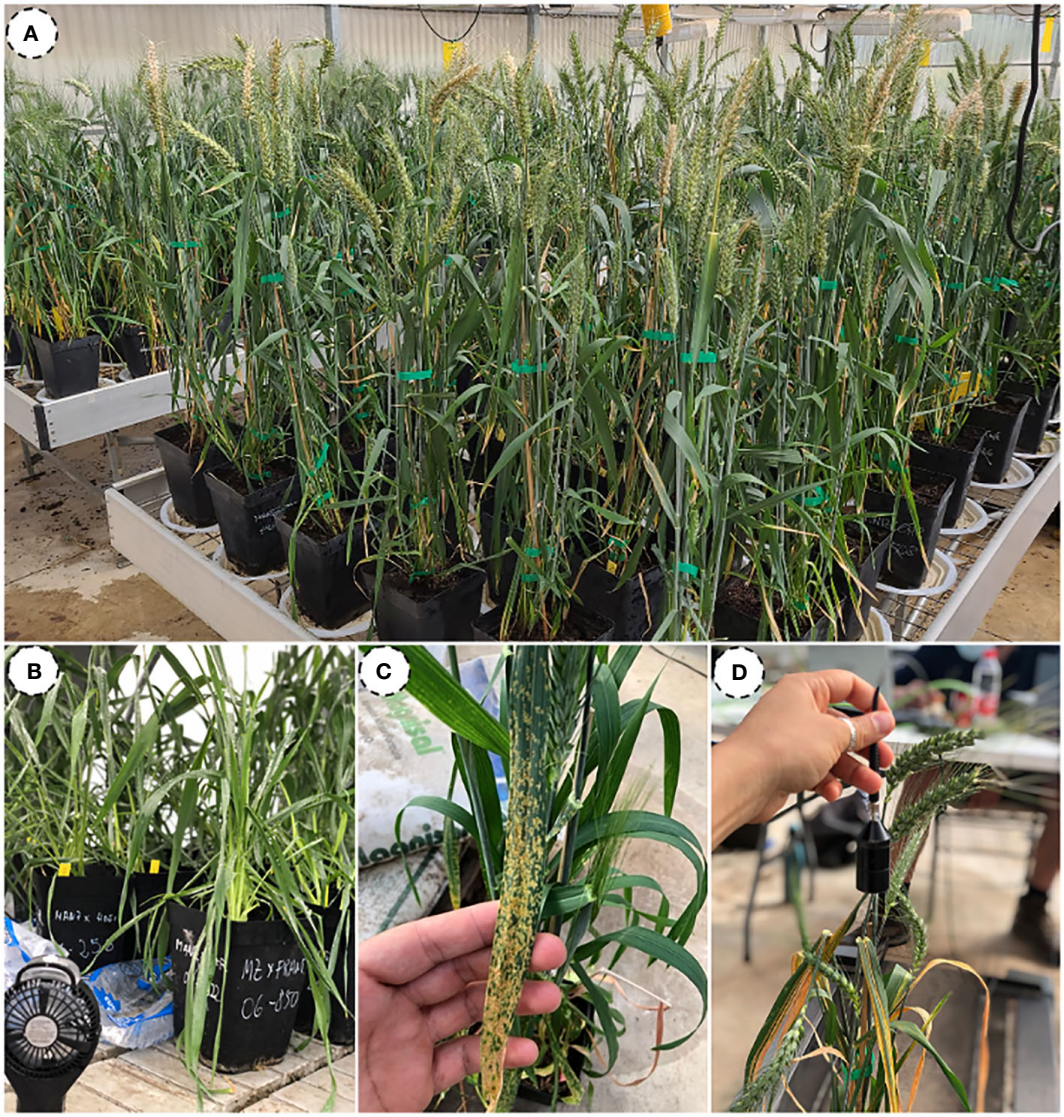
Illustration showing the experimental design **(A)**, the inoculation process **(B)**, a detailed view of leaves with yellow rust **(C)**, and the position of the spectroradiometer during the measurements **(D)**.

The field experiment was conducted in a greenhouse located at the School of Agricultural Engineering, University of Seville (37°21′9″ N, 5° 56 ′ 10.5 ′ W; Datum: WGS84), Spain. The study was conducted on spring wheat (*Triticum aestivum* L.) cultivated during the 2020/2021 growing season. The experiment included three cultivars of durum wheat, namely ‘Don Ricardo’, ‘Kiko Nick’, and ‘Amilcar’, as well as three cultivars of bread wheat, specifically ‘Conil’, ‘Califa’, and ‘Arthur Nick’. These cultivars were arranged in a randomized design with six replicates for each cultivar. Half the pots were inoculated with rust races to have healthy and infected pots. Pots of bread wheat were inoculated with yellow rust (*Puccinia striiformis* f. sp. *tritici.)*, and pots belonging to durum wheat were inoculated with brown rust, also called leaf rust (*Puccina triticina)*. The inoculation occurred on days 87 and 94 after seeding (DAS) for bread and durum wheat, respectively (**Figure 1**).

In addition to the visual score evaluation, each pot was subject to three spectral measurements captured at a distance of about 0.15 meters from the plant. Before the visibility of symptoms, the spectral signature was derived from the average of the canopy. However, once the pustules became completely visible, measurements were explicitly taken from the affected areas using a portable spectroradiometer. The sensor used was the spectroradiometer (UNISPEC-DC, PP-systems, Inc., Amesbury, MA, USA), which allows the measurement of reflectance from two optical fibres, channels A and B. One channel records the incident radiation, while the other records the reflected radiation. Each channel includes a photodiode detector that covers a spectral region ranging from 310 to 1100 nm. The sensor offers a spectral resolution between 3.1 and 3.4 nm. A white reference (99% reflectance Spectralon panel) calibrated the spectroradiometer. Hyperspectral data were collected around noon under completely sunny conditions, with data collection performed for each pot at intervals of 3-4 days. Seven measurements were made on pots inoculated with yellow rust on DAS 87, 94, 98, 101, 105, 108, and 112, and six measurements were made on pots inoculated with brown rust on DAS 94, 98, 101, 105, 108, and 112.

### 2.2 Data preprocessing

For each wavelength ( λ) , the spectral reflectance ( $R_{\lambda })\;$ was calculated following Equation 1:


(Equation 1) $$R_{\lambda }(\%)=\frac{L_{\lambda }^{r}}{L_{\lambda }^{i}}\times 100$$

where $L_{\lambda }^{r}$ denotes the spectral radiance the crop surface reflects in wavelength λ and $L_{\lambda }^{i}$ the spectral radiance the crop surface receives in wavelength λ .

The spectral signature of each pot was obtained by calculating the mean of the three measurements taken. This resulted in 36 spectral signatures for bread wheat and 36 for durum wheat. These spectra were classified into three groups: ‘Healthy’ (H) for non-inoculated plants, ‘Asymptomatic Leaf’ (AL) for inoculated plants without visible symptoms, and ‘Symptomatic Leaf’ (SL) for inoculated plants displaying visual symptoms. The selection of these categories was intentional, serving as target variables for prediction. Each category was meticulously crafted to include a diverse range of instances, thereby facilitating the development of accurate and robust predictive models. Subsequently, the spectra underwent standardization using the Scikit-learn package version 1.2.2 (Pedregosa et al., 2011), scaling the values from 0 to 1. Machine learning estimators often need standardization procedures as they perform optimally when features exhibit an approximately normal distribution. Following standardization, the Savitzky-Golay algorithm (Dópido et al., 2012) was applied with the following parameters: a window frame length of 11, polynomial order of 4, and the first derivative.

To mitigate the substantial variance in the quantity of data entries across categories, SMOTE (Synthetic Minority Over-sampling Technique) was utilized to augment the available data. The SMOTE technique is grounded in oversampling the minority class, thereby generating synthetic data for each data point within this underrepresented class. To generate these synthetic data points, the feature vector of the sample is subtracted from its nearest neighbour. This difference is then multiplied by a random number between 0 and 1 and added to the feature vector. Thus, synthetic data points are generated along the linear segments connecting any or all nearest neighbours, chosen randomly and based on the required oversampling. This study employed a random state of 888 to ensure reproducibility. **Table 1** illustrates the data points for each category before and after applying the SMOTE technique. Furthermore, the proportion of actual data within each category is provided after the SMOTE procedure.

**Table 1:** Comparative data on bread wheat and durum wheat cultivars.

Categories	Bread wheat		Durum wheat	
	Number of actual data	Actual data (%)	Number of actual data	Actual data (%)
**H**	125	100	107	100
**AL**	44	35.2	54	50.46
**SL**	64	51.2	36	33.64

The table presents the number and percentage of actual data for each category: Healthy (H), Asymptomatic Leaf (AL), and Symptomatic Leaf (SL).

Various dataset processing techniques were utilized to assess the influence of synthetic data generated by SMOTE on the development of prediction models. Throughout all scenarios, the H category was solely composed of actual data. The distinct processing methods are as follows:

• No SMOTE was applied; no synthetic data was introduced.
• SMOTE was applied to the entire data set: synthetic data were introduced into the training, testing, and second validation sets.
• SMOTE applied to the training set only: The testing and second validation sets consisted exclusively of actual data.

### 2.3 Training of ML models

**Figure 2:**
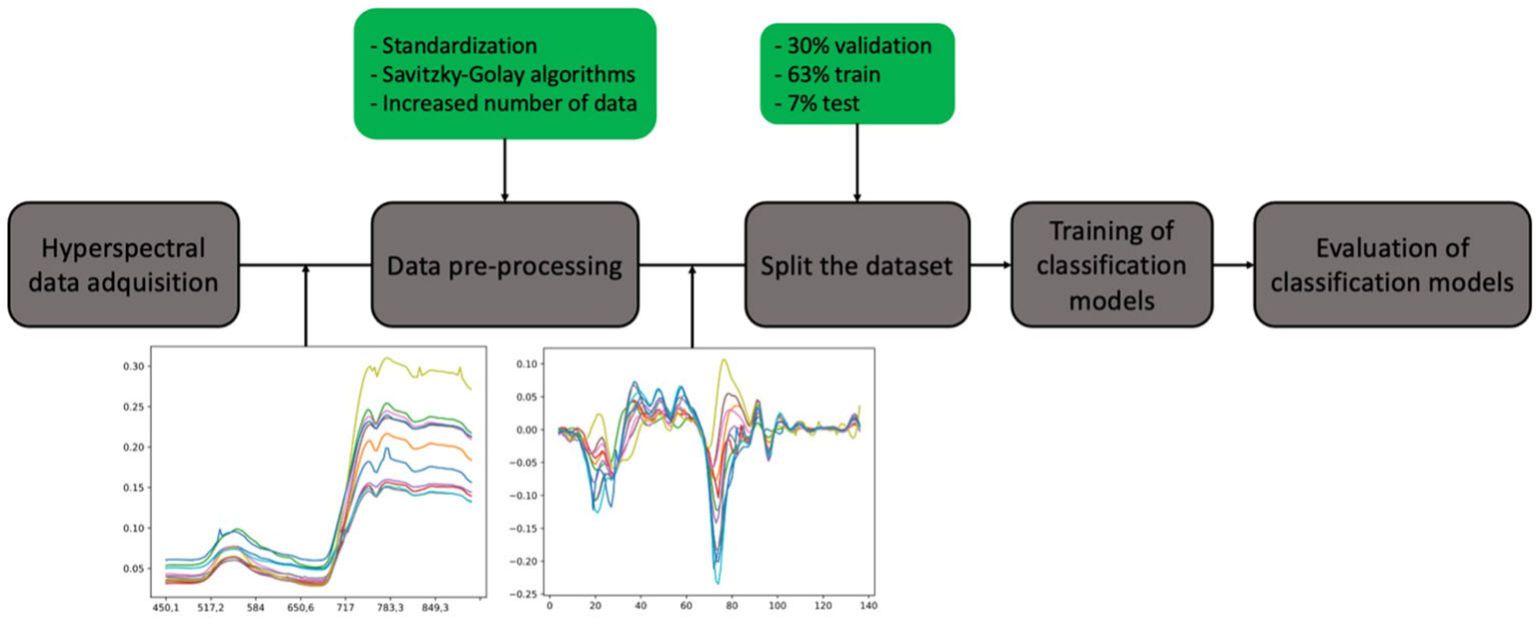
Workflow of hyperspectral data processing for disease classification in wheat. The process begins with hyperspectral data acquisition, followed by data pre-processing, including standardization and Savitzky-Golay filtering, with increased data points. The dataset is split into 63% for training, 30% for validation, and 7% for testing. Subsequently, the training of classification models is conducted, culminating in the evaluation of the model’s performance.

After the preprocessing step, the dataset was split into three parts: 30% for validation, 63% for training, and 7% for testing the models. The flowchart (**Figure 2**) provides the workflow associated with the different stages involved in disease detection.

Determining the best ML model for classification purposes is a challenging task, and often, the optimal decision is made through trial and error (Jagtap et al., 2022). This study implemented four models with the scikit-learn library: ANN, SVM, RF, and GNB. The RF and GNB models were configured with default parameters. A second-degree polynomial kernel was employed for the SVM model, with an independent term value of 2 in the kernel function.

The development of the ANN entailed utilizing RandomizedSearchCV to optimize the parameter settings. A total of 50 interactions were performed, with a random state set to 42. The parameters considered during the optimization process were alpha, hidden layer sizes, and learning rate init. For yellow rust, alpha was set to 0.0001, hidden layer sizes were 20 and 20, and the learning rate was set to 0.001. Conversely, alpha was set to 0.1 for brown rust, the hidden layer size was 30, and the learning rate was set to 0.01. The solver employed for the yellow rust dataset was Adam, while for brown rust, LBFGS was selected due to its better suitability for the data structure. All other parameters retained the default configuration of Scikit Learn. The ANN models developed using the dataset without the SMOTE application served as a reference because they achieved the highest accuracy results (see **Table 2**).

**Table 2:** F1-scores achieved by the SVM (Support Vector Machine) model for wheat disease classification are presented for the categories Healthy (H), asymptomatic leaf (AL), and Symptomatic Leaf (SL) across datasets for both yellow rust and brown rust.

Categories	Yellow rust			Brown rust		
	Without SMOTE	SMOTE	SMOTE on trainning	Without SMOTE	SMOTE	SMOTE on trainning
**H**	0.75	0.77	0.68	0.70	0.64	0.74
**AL**	0.54	0.87	0.50	0.46	0.75	0.54
**SL**	0.67	0.89	0.63	0.29	0.94	0.44

The table compares model performance without using SMOTE, with SMOTE, and with SMOTE applied during the training phase.

### 2.4 Matrics for model evaluation

The data processing was conducted using Google Collaboratory, which provides the necessary Python environment and libraries for data analysis and visualization. Regarding statistical assessment, the classification models were compared based on their network classification accuracy. Accuracy (Equation 2) quantifies the percentage of instances in which the model has made correct predictions, and it is defined as follows:


(Equation 2) $$\text{Accuracy}=\frac{\text{Number of correct predictions}}{\text{Total number of predictions}}$$

For each category with balanced data, evaluation was performed using the F1_score (Equation 3) derived from the confusion matrix, and it is defined as:


(Equation 3) $$\text{F}1\_\text{score}=\frac{2\text{x precision x recall}}{\text{precision}+\text{recall}}$$

where precision (Equation 4) and recall (Equation 5) are defined as follows:


(Equation 4) $$\text{Precision}=\frac{\text{TP}}{\text{TP}+\text{FP}}$$

(Equation 5) $$\text{Recall}=\frac{\text{TP}}{\text{TP}+\text{FN}}$$

,where TP represents True Positive, FP stands for False Positive, and FN represents False Negative. For models trained with imbalanced categories, precision was employed for their evaluation.

## 3 Results

### 3.1 Spectral reflectance analysis

**Figure 3:**
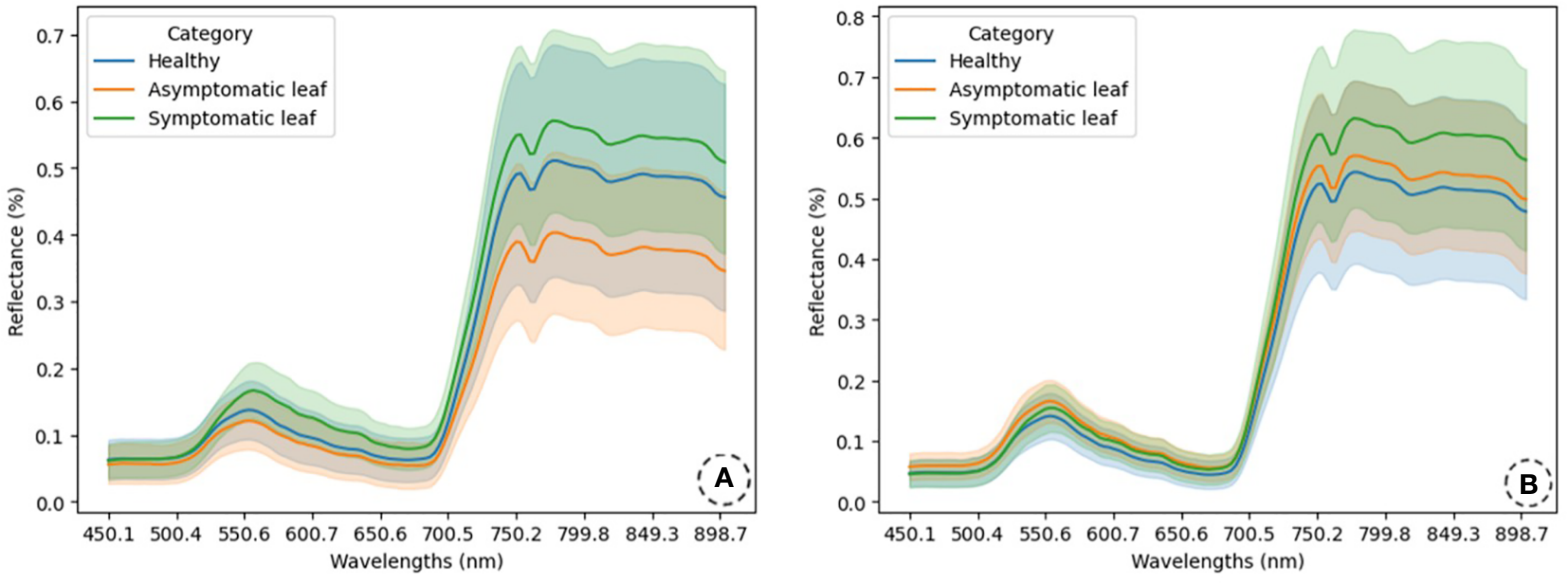
Reflectance spectra are presented for healthy (H), asymptomatic (AL), and symptomatic leaf (SL) categories, illustrating the spectra of wheat leaves affected by yellow rust **(A)** and brown rust **(B)**. Mean reflectance values and standard deviations have been computed for these predefined categories. The spectra are displayed in an unnormalized format.

As can be observed in **Figure 3**, the mean values can vary between categories, especially for yellow rust (**Figure 3A**). However, in brown rust (**Figure 3B**), the mean values exhibit a higher degree of overlap between categories. Overall, the mean reflectance values obtained for brown rust are higher than for yellow rust. In both cases, the most significant overlap occurs in the visible spectrum region, although it also occurs between the “H” and “AL” categories for brown rust. Both types of rust show considerable standard deviations, leading to significant overlap across all categories. To address this issue, specific classification models have been developed for each rust type to enhance accuracy in categorization. Notably, for yellow rust, the mean value of the healthy category exceeds that of the asymptomatic leaf category, while the reverse is true for brown rust.

Upon close examination of each plot at the rust level, it is observed that for yellow rust (**Figure 3A**), the category with the lowest mean reflectance value is the asymptomatic leaf, followed by the healthy category. In contrast, the symptomatic leaf category displays the highest mean reflectance value. This trend remains consistent in the visible spectrum (400-700 nm) and NIR regions (700-800 nm). All categories exhibit a standard deviation wide enough to cause overlap, although the healthy category displays the highest variability. In the visible spectrum region, the similarity in mean reflectance values between the healthy and asymptomatic leaf categories is noteworthy, with the symptomatic category achieving a higher mean value than both. In the NIR region, the mean reflectance difference increases between the healthy and asymptomatic leaf categories while it decreases between the healthy and symptomatic leaf categories.

**Figure 3B** presents the mean reflectance and standard deviation values for the various categories of wheat leaves infested with brown rust. The mean reflectance values and standard deviations are similar across the categories in the visible spectrum region. However, in the NIR region, there is a noticeable increase in the mean reflectance value for the symptomatic leaf category compared to the others, indicating that the spectral signatures of H and asymptomatic plants are very similar, which presents challenges in early detection. Similar to yellow rust, the data obtained for brown rust also exhibit significant standard deviations, resulting in an overlap among categories.

### 3.2 Models’ performance

The classification models were constructed using the training dataset, encompassing labelled data from all wheat varieties. The models were fine-tuned using the validation dataset. Subsequently, the developed models were tested for performance using the test set, comprising spectral data from all varieties within each wheat type. The corresponding accuracy (%) of each model used in this study, based on their respective datasets, is presented in **Table 3**.

**Table 3:** Performance comparison of Machine Learning (ML) models for yellow and brown rust classification.

Models	Yellow rust			Brown rust		
	Without SMOTE	SMOTE	SMOTE on training	Without SMOTE	SMOTE	SMOTE on training
**ANN**	65.71	76	60	57	76.3	55
**SVM**	68.6	85	62.86	58	78.35	63
**RF**	70	81.5	68.5	53	73.2	55
**GNB**	64	61	57	37	60	38

The table presents the accuracy percentages of Artificial Neural Networks (ANN), Support Vector Machines (SVM), Random Forests (RF), and Gaussian Naive Bayes (GNB) with and without the application of Synthetic Minority Over-sampling Technique (SMOTE) during training.

The results show that the model’s accuracy is consistently higher for classifying yellow rust than brown rust. Among the models, the GNB model displayed the least accuracy in both yellow and brown rust contexts. Consequently, our analysis will primarily concentrate on the outcomes achieved by the ANN, SVM, and RF models.

The SVM model obtained the highest accuracy for the dataset where SMOTE was not applied, followed by the ANN model for brow rust and the RF model for yellow rust. Similarly, in cases where the dataset was augmented using SMOTE, the highest accuracy values were obtained by SVM models. The accuracy achieved in this dataset is the highest among all models compared to the results obtained in the remaining datasets. Furthermore, these models also excel in the dataset where the SMOTE algorithm was exclusively applied during training. Nevertheless, it is noteworthy that the accuracy obtained in the SMOTE dataset during training decreased compared to the datasets where SMOTE was and was not applied for all models. However, the exception to this trend is observed for the SVM and RF models in the case of brown rust. In the SVM model, the accuracy of the model trained with the original dataset increased by five percentage points when SMOTE was applied in training. For the RF model, this increase was two percentual points.

**Table 4** shows the F1-scores achieved by the ANN model for yellow and brown rust prediction. Regarding yellow rust, it can be observed that in the dataset without SMOTE, the “H” category, characterized by a more substantial number of data points, achieved higher values. In contrast, the “AL” category displayed the lowest value. It is worth noting that this category was composed of fewer data than the others. In the dataset where the algorithm was fully implemented, notable enhancements were observed in the “AL” and “SL” categories, which incorporated synthetic data. However, the “H” category, comprised solely of actual data, obtained a lower score than the dataset where SMOTE was not applied. Conversely, in the dataset where SMOTE was only applied to the training dataset, it was observed that the “H” category maintained an outcome similar to that of the dataset with complete SMOTE application and a decrease relative to the original dataset. However, the “SL” and “AL” categories obtained similar and slightly higher F1-scores than the dataset where the SMOTE algorithm was not applied.

**Table 4:** F1-scores achieved by the ANN (Artificial Neural Network) model for wheat disease classification are presented for the categories Healthy (H), asymptomatic leaf (AL), and Symptomatic Leaf (SL) across datasets for both yellow rust and brown rust.

	Yellow rust			Brown rust		
Categories	Without SMOTE	SMOTE	SMOTE on training	Without SMOTE	SMOTE	SMOTE on training
H	0.73	0.63	0.65	0.66	0.66	0.67
AL	0.45	0.77	0.50	0.47	0.81	0.61
SL	0.62	0.87	0.59	0.37	0.82	0.63

The table compares model performance without using SMOTE, with SMOTE, and with SMOTE applied during the training phase.

A similar trend is observed for brown rust as for yellow rust. The category “H” results were consistent across all three datasets. In the categories “AL” and “SL,” higher F1-scores were obtained in the dataset where SMOTE was fully applied. In contrast to the trend observed in yellow rust, in the dataset where SMOTE was only used in the training, categories SL and AL increased their accuracy by 26 and 14 points, respectively, compared to the dataset where SMOTE was not applied. The F1-scores obtained by the SVM models show behaviour similar to that of the ANN model (**Table 2**). The “H” category demonstrates consistent performance across all three datasets with slight variations. For the “AL” and “SL” categories, a notable enhancement is observed when SMOTE is applied to the entire dataset, contrasting the performance of the non-SMOTE dataset. However, in the dataset where SMOTE was solely used during training, the accuracy obtained decreases by 3-4 percentual points for yellow rust and increases for brown rust. In the latter case, the 15-point increase in the “SL” category is worth noting compared to the original dataset.

**Table 5** displays the F1-scores results of the RF model. In the case of yellow rust, the “H” category maintains consistency across all datasets, with a slight advantage in the non-SMOTE dataset. The “AL” category shows improvement with SMOTE applied during training, while “SL” remains unchanged. A similar pattern is observed for the “H” category in the context of brown rust. Interestingly, the dataset containing actual data yielded the lowest values for “AL” and “SL”, but the application of SMOTE during training increased their values by 12 and 20 points, respectively.

**Table 5:** F1-scores achieved by the RF (Random Forest) model for wheat disease classification are presented for the categories Healthy (H), asymptomatic leaf (AL), and Symptomatic Leaf (SL) across datasets for both yellow rust and brown rust.

Categories	Yellow rust			Brown rust		
	Without SMOTE	SMOTE	SMOTE on training	Without SMOTE	SMOTE	SMOTE on training
**H**	0.78	0.70	0.73	0.66	0.59	0.64
**AL**	0.32	0.84	0.50	0.34	0.71	0.46
**SL**	0.71	0.88	0.74	0.17	0.89	0.37

The table compares model performance without using SMOTE, with SMOTE, and with SMOTE applied during the training phase.

When comparing the results obtained for each model, it can be observed that the best accuracies are achieved by the models that used datasets augmented with the SMOTE algorithm. However, the presence of synthetic data in the test dataset may raise concerns about the reliability of the results. Regarding the dataset for the category consisting solely of actual data, RF was the best model for yellow rust classification. The SVM model performed better for brown rust when the same dataset was used.

The SVM model achieved the highest F1-score for yellow rust in the “AL” category. In the case of brown rust, the best model was ANN for the same category. Conversely, the highest accuracy for the “AL” category in yellow rust was found in the original dataset, and for brown rust, it occurred in the dataset where SMOTE was applied during training. Finally, the best F1-score for the “SL” category in yellow rust was achieved by the RF model, and for brown rust, it was the SVM model, both using the dataset with SMOTE applied during training.

### 3.3 Confusion matrix

**Figure 4:**
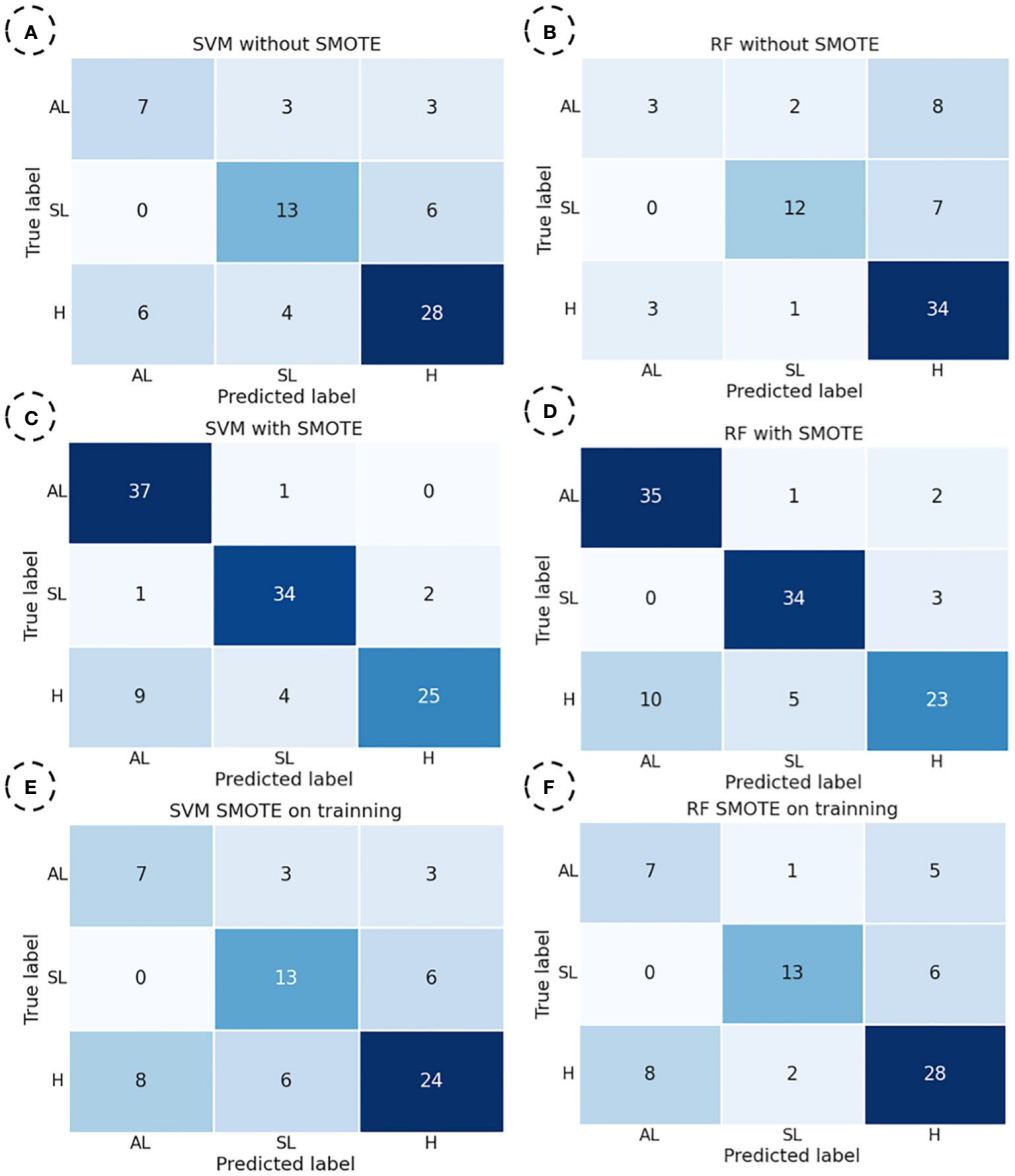
Confusion matrices were obtained by SVM (Support Vector Machine) and RF (Random Forest) models to predict “H”, “SL”, and “AL” yellow rust categories. SVM for the dataset without SMOTE **(A)**, RF for the dataset without SMOTE **(B)**, SVM for the dataset with SMOTE **(C)**, RF for the dataset with SMOTE **(D)**, SVM for the dataset with SMOTE on training **(E)**, RF for the dataset with SMOTE on training **(F)**.

For the set of confusion matrices shown in **Figure 4**, it was observed that both models had a similar total number of errors in the dataset where SMOTE was not applied. However, the number of classification errors by categories differs significantly between the two models. In the SVM model, the category with the highest number of errors was “H” particularly when distinguishing it from the “AL” category. Notably, there were many mistakes in classifying the “SL” and “H” categories. On the other hand, in the RF model, there is a drastic decrease in the error rate for classifying the “H” category, representing an improvement compared to the SVM model. However, an increase in misclassifications in the “AL” and “SL” categories was observed, especially when distinguishing them from the “H” category. In the dataset where SMOTE was applied entirely, both models exhibited the highest number of correct predictions in the categories “AL” and “SL,” which included synthetic data. However, most errors occurred in the category “H”, consisting entirely of actual data, particularly in distinguishing between “H” and “AL”. Notably, the number of errors in this distinction is higher in this dataset than in the original data.

Finally, in the dataset where SMOTE was exclusively applied to the training set, a similar pattern was observed compared to the dataset without SMOTE. The category with the highest number of correct predictions was “H,” which showed better results with RF than SVM. For this category, the SVM model exhibited a more significant number of incorrect predictions with the “SL” category. On the other hand, for the “AL” category in the SVM model, the number of errors was balanced with the “SL” and “H” categories. However, in the RF model, it is observed that most incorrect predictions were made mainly concerning the “H” category.

Based on the data obtained in **Figures 4A, B, E, F**, it is evident that the RF model is better at classifying the predominant category “H” consisting exclusively of real data. All models exhibit similar behaviour regarding the “SL” category, whose spectral characteristics differ the most from the other categories. Finally, for the “AL” category, it is noteworthy that both SVM and RF models perform well when SMOTE is applied during training. However, they misclassify instances differently, with the RF model standing out. This is attributed to its ability, within the margin of error, to more accurately approximate two categories with similar spectral characteristics, namely “AL” and “H”.

**Figure 5:**
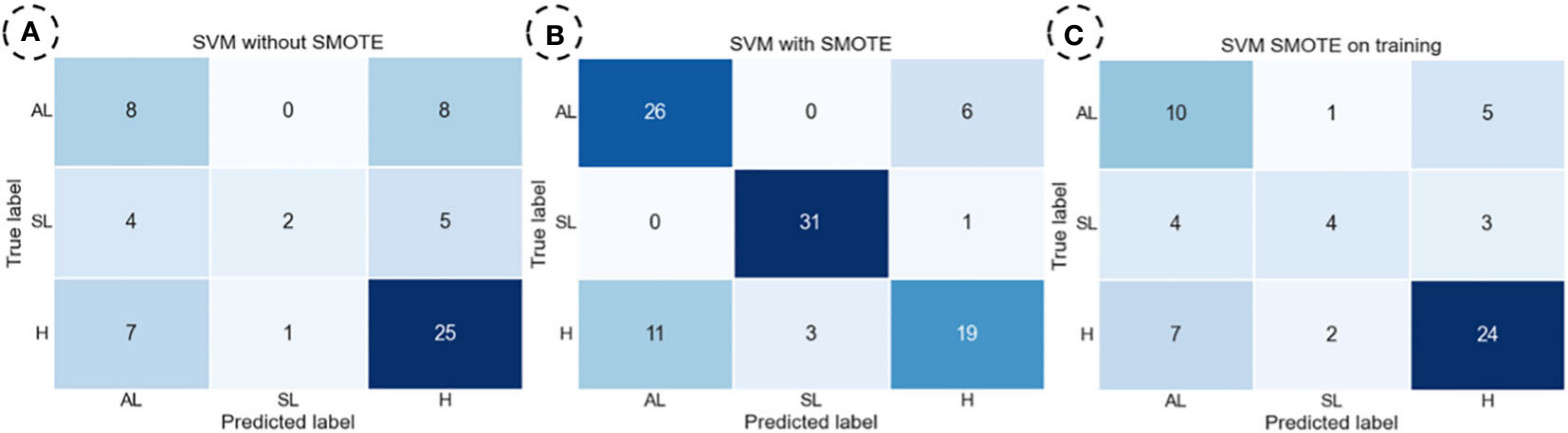
The SVM (Support Vector Machine) model obtained confusion matrices to predict Healthy (H), Symptomatic Leaf (SL), and Asymptomatic (AL) brown rust categories. SVM for the dataset without SMOTE **(A)**, SVM for the dataset with SMOTE **(B)**, and SVM for the dataset with SMOTE on training **(C)**.

The confusion matrices obtained with the SVM model for each dataset with the highest accuracy for brown rust are displayed in **Figure 5**.

For the set of confusion matrices shown in **Figure 5**, it was observed that, in the dataset in which SMOTE was not applied, the category with the highest number of correct predictions is ‘H.’ Within this category, it is noteworthy that the highest number of errors was made with the ‘AL’ category. The same pattern is repeated for the ‘AL’ category, with all incorrect predictions made with the ‘H’ category. The ‘SL’ category showed the most significant errors, evenly distributed among the remaining categories.

The same behaviour was observed for yellow rust in the dataset where SMOTE was applied entirely. Finally, the trend observed for the ‘H’ category without data augmentation repeats itself in the dataset where SMOTE was exclusively applied to the training set. However, for the ‘SL’ and ‘AL’ categories, there is a slight increase in the number of correct predictions, and the error ratio remains consistent compared to the original dataset. Therefore, this latter model demonstrates the highest efficiency in category distinction, although it also shows notable deficiencies in classifying the ‘SL’ category.

## 4 Discussion

This study examines the spectral reflectance signatures for three different disease categories. It explores the application of the SMOTE algorithm across various hyperspectral datasets for predicting wheat rust, specifically focusing on its impact on model accuracy and F1-scores.

Regarding leaf rust classification, the present study yields results similar to those obtained by Ruan et al. (2021), who achieved an accuracy of 86.2% using an SVM model to classify healthy and rust-infected wheat leaves. While they also employed SMOTE to balance the data, they did not examine its effects. However, our findings demonstrate a significant improvement in model performance by applying the SMOTE algorithm. Specifically, we observed accuracy improvements ranging from 16% to 20% for yellow rust and 11% to 20% for brown rust when SMOTE was applied across the entire dataset. These results align with previous studies, such as those by Uğuz and Uysal (2021) and Singh and Arora (2020), which utilized a dataset of 3400 hyperspectral images to distinguish between two diseases and healthy plants across three categories. Nevertheless, our study offers a detailed analysis, particularly concerning the category comprised solely of original data, where no accuracy improvement was noted. This highlights the complex effects of data augmentation techniques like SMOTE on model accuracy. Similarly, Su et al. (2019) reported a 3.36% improvement in model performance when comparing outcomes on imbalanced versus balanced standard datasets, underscoring the beneficial impact of data-balancing techniques.

In contrast to our findings, Singh and Arora (2020) reported an increase in overall accuracy across all categories when applying the SMOTE algorithm, with 75% of their dataset comprising synthetic data. This discrepancy highlights the varied outcomes that can occur based on the dataset’s composition, particularly the proportion of synthetic data introduced. This variability in results highlights the complex relationship between dataset characteristics and the efficacy of data augmentation techniques, prompting a more thorough investigation into the factors influencing model performance. Furthermore, our study adds to the extensive literature on leveraging the complete spectrum of spectroradiometers for plant disease detection. Works such as Naidu et al. (2009) and Khosrokhani and Nasr (2022) have demonstrated the potential of combining spectral data with machine learning models, yielding high accuracy rates. Our findings are consistent with these studies, particularly in revealing more significant classification errors for categories with analogous spectral characteristics.

Sun et al. (2024) utilized SMOTE to assess the severity of peanut blight. They concluded that while SMOTE serves as a valuable approach for tackling data imbalance, it is important to mention that SMOTE generates synthetic samples containing noise. This observation could explain why, in our study, no notable differences were found in the “H” class upon applying the algorithm. The spectra of healthy leaves closely resemble those generated by SMOTE. However, the algorithm exhibited better performance for the other classes, as it is more common to encounter noise in infected leaves, primarily due to pustules.

In conclusion, our research enhances the understanding of the role of data augmentation in machine learning for plant disease detection. It underscores the importance of large, diverse datasets and the careful consideration of the balance between actual and synthetic data. The choice of machine learning models should be tailored to the specific characteristics of the dataset and the disease under investigation. This study contributes to academic knowledge and holds practical implications in agricultural technology, especially in developing robust, accurate systems for early disease detection and management.

## 5 Conclusion

This study investigated the efficacy of various ML models in detecting yellow and brown rust in wheat crops using hyperspectral data, emphasizing the role of SMOTE in enhancing model accuracy. SMOTE significantly improved model accuracy, particularly in training datasets, especially for minority categories with synthetic data. However, this might affect real-world applicability due to potential accuracy distortion. The RF model showed 70% accuracy for yellow rust using only actual data. The SVM model achieved 63% accuracy for brown rust when SMOTE was applied to the training set, highlighting these models’ ability to discern features effectively. However, similarity in spectral characteristics between specific categories, like ‘H’ and ‘AL’, posed challenges. The application of SMOTE generally decreased the performance of the ‘H’ class in both RF and SVM models. Still, it improved accuracy for minority classes ‘AL’ and ‘SL’, achieving 61% accuracy for the ‘AL’ category in brown rust detection. These findings underline the importance of data augmentation for enhancing category-specific accuracy and advocate for further research into data processing and augmentation techniques to refine ML model performance in hyperspectral data analysis.

## Data availability statement

The raw data supporting the conclusions of this article will be made available by the authors, without undue reservation.

## Author contributions

CC: Methodology, Software, Writing – original draft. OA: Data curation, Formal analysis, Investigation, Writing – original draft. JR: Data curation, Writing – original draft. GE: Conceptualization, Funding acquisition, Project administration, Writing – review & editing. MP: Conceptualization, Funding acquisition, Project administration, Writing – review & editing.
